# Migraine without aura: genome-wide association analysis identifies several novel susceptibility

**DOI:** 10.1186/1129-2377-14-S1-P18

**Published:** 2013-02-21

**Authors:** Boukje de Vries, Tobias Freilinger, Verneri Anttila, Rainer Malik, Mikko Kallela, Gisela M Terwindt, Patricia Pozo-Rosich, Bendik Winsvold, Dale R Nyholt, Willebrordus PJ van Oosterhout, Ville Artto, Unda Todt, Eija Hämäläinen, Jèssica Fernández-Morales, Mark A Louter, Mari A Kaunisto, Jean Schoenen, Olli Raitakari, Terho Lehtimäki, Marta Vila-Pueyo, Hartmut Göbel, Erich Wichmann, Cèlia Sintas, Andre G Uitterlinden, Albert Hofman, Fernando Rivadeneira, Axel Heinze, Erling Tronvik, Cornelia M van Duijn, Jaakko Kaprio, Bru Cormand, Maija Wessman, Rune R Frants, Thomas Meitinger, Bertram Müller-Myhsok, John-Anker Zwart, Markus Färkkilä, Alfons Macaya, Michel D Ferrari, Christian Kubisch, Aarno Palotie, Martin Dichgans, Arn MJM van den Maagdenberg

**Affiliations:** 1 Department of Human Genetics, Leiden University Medical Centre, Leiden, The Netherlands; 2 Institute for Stroke and Dementia Research, Klinikum der Universität München, Munich, Germany; 3 Department of Neurology, Klinikum der Universität München, Munich, Germany; 4 Wellcome Trust Sanger Institute, Wellcome Trust Genome Campus, Cambridge, UK; 5 Institute for Molecular Medicine Finland (FIMM), University of Helsinki, Helsinki, Finland; 6 Department of Neurology, Helsinki University Central Hospital, Helsinki, Finland; 7 Department of Neurology, Leiden University Medical Centre, Leiden, The Netherlands; 8 Department of Neurology, Vall d'Hebron University Hospital, Universitat Autònoma de Barcelona, Barcelona, Spain; 9 Headache Research Group, Vall d'Hebron Research Institute, Universitat Autonoma de Barcelona, Barcelona, Spain; 10 Department of Neurology, Oslo University Hospital and University of Oslo, Oslo, Norway; 11 Neurogenetics Laboratory, Queensland Institute of Medical Research, Brisbane, Australia; 12 Institute of Human Genetics, University of Ulm, Ulm, Germany; 13 Department of Psychiatry, Leiden University Medical Centre, Leiden, The Netherlands; 14 Folkhälsan Research Center, Helsinki, Finland; 15 Headache Research Unit, Department of Neurology and Groupe Interdisciplinaire de Génoprotéomique Appliquée (GIGA)-Neurosciences, Liège University, Liège, Belgium; 16 Department of Clinical Physiology, University of Turku and Turku University Central Hospital, Turku, Finland; 17 Department of Clinical Chemistry, Tampere University Hospital and University of Tampere, Tampere, Finland; 18 Pediatric Neurology Research Group, Vall d'Hebron Research Institute, Barcelona, Spain; 19 Kiel Pain and Headache Center, Kiel, Germany; 20 Institute of Epidemiology, Helmholtz Center Munich, Neuherberg, Germany; 21 Department of Genetics, University of Barcelona, Barcelona, Spain; 22 Biomedical Network Research Centre on Rare Diseases (CIBERER), Barcelona, Spain; 23 Department of Internal Medicine, Erasmus Medical Center, Rotterdam, The Netherlands; 24 Department of Epidemiology, Erasmus University Medical Center, Rotterdam, The Netherlands; 25 Department of Neuroscience, Norwegian University of Science and Technology, Trondheim, Norway; 26 Department of Public Health, University of Helsinki, Helsinki, Finland; 27 Department of Mental Health and Alcohol Research, National Institute for Health and Welfare, Helsinki, Finland; 28 Institut de Biomedicina de la Universitat de Barcelona (IBUB), Barcelona, Spain; 29 Institute of Human Genetics, Helmholtz Zentrum München, Neuherberg, Germany; 30 Institute of Human Genetics, Klinikum rechts der Isar, Technische Universität München, Munich, Germany; 31 Max Planck Institute of Psychiatry, Munich, Germany; 32 The Broad Institute of MIT and Harvard, Boston, Massachusetts, USA; 33 Department of Medical Genetics, University of Helsinki, Helsinki, Finland; 34 Department of Medical Genetics, Helsinki University Central Hospital, Helsinki, Finland

## Introduction

Genome-wide association studies (GWAS) are a novel and promising method to study genetic susceptibility factors for common disorders, including migraine.

## Objective

Here we performed the first GWAS in migraine without aura (MO), which is the most common form of migraine.

## Methods

To identify common genetic variants for this migraine type, we analyzed genome-wide association data of 2,326 clinic-based German and Dutch patients and 4,580 population-matched controls. Loci with two or more SNPs with P-values < 1 x 10-5 were selected for follow-up in 2,508 Dutch, Spanish, Finnish and Norwegian patients and 2,652 controls.

## Results

Meta-analysis of the discovery and replication data yielded four genome-wide significant (P < 5 x 10-8) MO susceptibility loci in or nearby MEF2D, PHACTR1, ASTN2 and TGFBR2. In addition, SNPs in two loci (in or near TRPM8 and LRP1) that were previously identified in a GWAS on population-based migraine were significantly replicated in our clinic-based MO cohort.

## Conclusion

This study reveals the first susceptibility loci for migraine without aura, thereby expanding our knowledge of this debilitating neurological disorder.

